# Biology and therapy of inherited retinal degenerative disease: insights from mouse models

**DOI:** 10.1242/dmm.017913

**Published:** 2015-02

**Authors:** Shobi Veleri, Csilla H. Lazar, Bo Chang, Paul A. Sieving, Eyal Banin, Anand Swaroop

**Affiliations:** 1 Neurobiology-Neurodegeneration and Repair Laboratory, National Eye Institute, National Institutes of Health, Bethesda, MD 20892, USA.; 2 Molecular Biology Center, Interdisciplinary Research Institute on Bio-Nano Sciences, Babes-Bolyai-University, Cluj-Napoca, 400271, Romania.; 3 The Jackson Laboratory, Bar Harbor, ME 04609, USA.; 4 National Eye Institute, National Institutes of Health, Bethesda, MD 20892, USA.; 5 Center for Retinal and Macular Degenerations, Department of Ophthalmology, Hadassah-Hebrew University Medical Center, Jerusalem 91120, Israel.

**Keywords:** Mouse mutants, Photoreceptor, Retinal development, Retinal disease

## Abstract

Retinal neurodegeneration associated with the dysfunction or death of photoreceptors is a major cause of incurable vision loss. Tremendous progress has been made over the last two decades in discovering genes and genetic defects that lead to retinal diseases. The primary focus has now shifted to uncovering disease mechanisms and designing treatment strategies, especially inspired by the successful application of gene therapy in some forms of congenital blindness in humans. Both spontaneous and laboratory-generated mouse mutants have been valuable for providing fundamental insights into normal retinal development and for deciphering disease pathology. Here, we provide a review of mouse models of human retinal degeneration, with a primary focus on diseases affecting photoreceptor function. We also describe models associated with retinal pigment epithelium dysfunction or synaptic abnormalities. Furthermore, we highlight the crucial role of mouse models in elucidating retinal and photoreceptor biology in health and disease, and in the assessment of novel therapeutic modalities, including gene- and stem-cell-based therapies, for retinal degenerative diseases.

## Introduction

Light is a fundamental driver of daily functions and behavior in most organisms. In vertebrates, light is captured by photoreceptors in the retina and their output constitutes the major sensory input to the brain (Noback, 2005; Rodieck, 1998). In humans, vision is paramount for quality of life and the impairment of sight represents a highly incapacitating condition. Vision loss or dysfunction can be caused by obstruction of the light path to the neural retina or inability of the retina to detect and/or transmit light-triggered signals to the brain. In retinal degenerative diseases (RDDs), it is the latter that is largely responsible for incurable blindness due to dysfunction or death of photoreceptor cells. Genetic components determine the genesis and health of photoreceptors, and mutations that lead to structural and/or functional perturbations can eventually lead to blindness. RDDs can be broadly divided into monogenic (Mendelian) or multifactorial (complex) disorders. Several RDDs can be recognized in monogenic non-syndromic and syndromic forms (see Box 1 for a glossary of terms) with clinically distinguishable findings (Berger et al., 2010) (RetNet: https://sph.uth.edu/retnet/). For the purpose of this Review, we have focused on commonly observed Mendelian retinal diseases (Table 1). The most common multifactorial RDD is age-related macular degeneration (AMD). The readers are directed to excellent reviews on AMD (Cooke Bailey et al., 2013; Fritsche et al., 2014) for further information.

**Box 1. Glossary**

Achromatopsia: a clinical condition where the patient cannot see colors. The color spectrum is seen as shades of white and gray.
Bradyopsia: describes the condition when the visual system adapts more slowly than normal to low light levels.
Chaperonins: large proteins that promote proper folding of other proteins, prevent aggregation of mis-folded proteins and assist trafficking to the intended intracellular target(s).
Choroid: the vascular layer with connective tissue that lies underneath the retina and supplies oxygen and nutrients to the outer layers of the retina. It is also known as the choroidea.
Dark adaptation: the process in the dark of regaining full visual sensitivity and responsiveness of rod photoreceptors following a bleach of the visual pigment rhodopsin after exposure to light. In practical terms, the state of rod dark adaptation is normally tested after a 30- to 45-minute period in the dark by recording an electroretinogram response to a light flash stimulus.
Electroretinogram (ERG) ‘a’ and ‘b’ waves: the initial negative-going response is termed a-wave, which is generated by closure of the rod (or cone) light-gated channels, leading to hyperpolarization of the photoreceptor. The positive-going b-wave trace that follows the ‘a’ wave is produced by depolarization of the bipolar cells, which lie postsynaptically to the photoreceptors.
Fundus: the posterior inside of the eye that contains the sheet of retina neural tissue, which can be viewed by an ophthalmoscope or photographically. The fundus image can reveal the health or disease of the retina, including microvasculature and abnormalities in optic disc, macula and fovea.
Non-syndromic disease: a disease with clinical findings limited to a single tissue and/or function.
Primary cilium: the cilium is a microtubule-based organelle projecting from most eukaryotic cells. Primary cilia are non-motile with a 9+0 configuration of microtubule bundles but lack the central pair of microtubules present in the motile cilium.
Syndromic disease: a single disorder that affects multiple tissues or functions and causes pleiotropic clinical symptoms. For example, individuals with Bardet-Biedl syndrome have a primary cilia disease (ciliopathy) and exhibit diverse phenotypes including retinal degeneration, mental retardation, polycystic kidney and obesity.

**Table 1.**
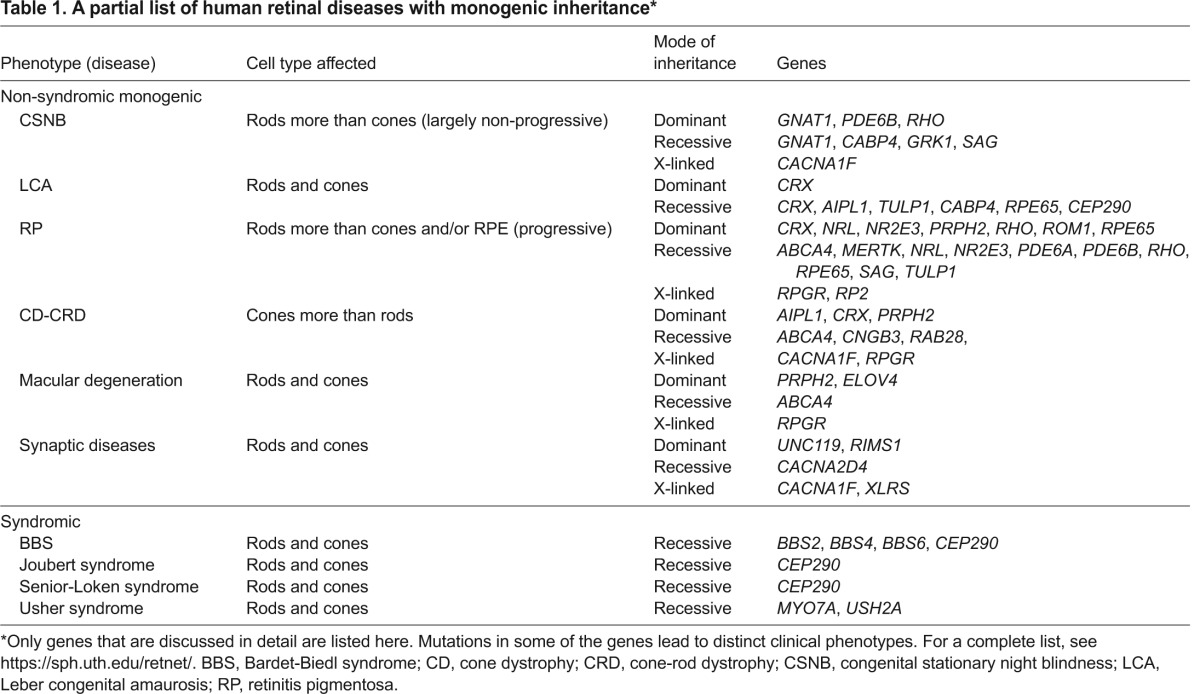
A partial list of human retinal diseases with monogenic inheritance*

During the last decade, genetic studies have provided tremendous insights into Mendelian forms of retinal diseases (Swaroop and Sieving, 2013), which afflict one in 2000–3000 individuals (Hartong et al., 2006). Retinitis pigmentosa (RP) is the most common form of inherited retinal degeneration, with a frequency of one in 3000–7000 individuals (Ferrari et al., 2011). Our understanding of molecular and genetic defects in Mendelian retinal blindness has improved tremendously in recent years (Wright et al., 2010), with the discovery of genetic defects in over 200 genes (RetNet: https://sph.uth.edu/retnet/). The advent of next-generation sequencing and better molecular diagnosis methods has enabled us to identify the genetic cause of inherited retinal disease in the majority of patients (Neveling et al., 2012; Ratnapriya and Swaroop, 2013). The major challenge now is to elucidate biological mechanisms of retinal disease pathogenesis, with the goal being the design of gene-based treatments.

The majority of genes associated with non-syndromic or syndromic retinal diseases influence photoreceptor development or function. In this Review, we focus on mouse models of monogenic retinal degeneration, where a genetic defect in a single gene is generally sufficient to cause disease. The ability of such models to assist in elucidating disease mechanisms was recognized very early by the identification of a naturally occurring nonsense mutation in the cGMP phosphodiesterase (PDE) subunit encoded by *Pde6b*, which causes rapid retinal degeneration in affected mice (Keeler, 1924; Pittler and Baehr, 1991; Sidman and Green, 1965). As molecular genetic methods improve, naturally occurring models have been augmented by genetically engineered mouse models that have been immensely valuable in advancing our understanding of retinal development and degeneration. These models provide fundamental insights into biological pathways and often display phenotypes that are similar to clinical manifestations of the corresponding disease in humans, providing an opportunity to decipher mechanisms of disease pathology as well as develop therapies. However, the progress in the generation and characterization of mouse retinal disease models has been relatively slow despite the rapid pace of disease gene discovery during the last decade. With the advent of new technologies, such as ‘clustered regularly interspaced short palindromic repeats’ (CRISPR) (Wang et al., 2013; Yang et al., 2013), we should be able to quickly produce mouse mutants with single- or even multi-gene defects.

In the following sections, we begin by describing the structure and function of the retina, followed by the genetics of hereditary retinal degeneration and discussion of the most relevant mouse models for RDDs. Next, we outline current techniques used for evaluating retinal degeneration in humans and mice, followed by a description of specific forms of RDDs caused by perturbations in photoreceptor development, intracellular trafficking, cilia biogenesis, phototransduction and synaptic function. RDDs associated with retinal pigment epithelium (RPE) dysfunction are also addressed. Finally, we discuss the importance of mouse models of RDDs in discovering novel therapeutic interventions for blinding retinal diseases.

## Retina structure and function

**Fig. 1.**
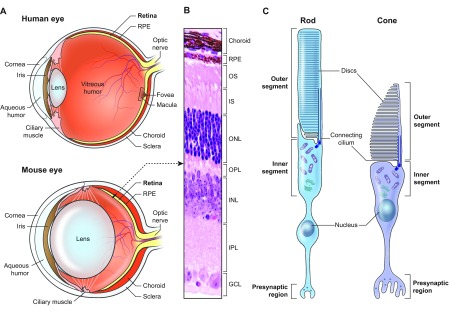
**Structure of the human and mouse eye.** (A) Schematic cross-sections of the human and mouse eye. Light is focused by optical elements (such as cornea and lens) on the neural retina at the back of the eye. The central cone-only region of the human retina is called the fovea and is responsible for high resolution. The region surrounding the fovea is termed macula and contains higher density of cones compared with the peripheral retina. The area of human retina is ~1094 mm^2^, with the macula and fovea being ~3 and 1.5 mm^2^, respectively (http://webvision.med.utah.edu). The total number of rods and cones in the human retina are 120 million and 6 million, respectively. The highest density of cones is at the center of the fovea (~161,900/mm^2^), which has no rods. The mouse retina lacks a distinct fovea and/or macula. The retinal pigment epithelium (RPE) monolayer separates the choroidal blood supply from the photoreceptors and is crucial for visual function. The lens is much larger in mouse than humans relative to the eye size. (B) Photograph of a mouse retinal section stained with hematoxylin and eosin, indicating different cellular layers. The outer nuclear layer (ONL) contains photoreceptor cell bodies, from which the inner segment (IS) and outer segment (OS) extend towards the RPE. The inner nuclear layer (INL) includes amacrine, bipolar and horizontal neurons, whereas ganglion cells, axons of which form the optic nerve, reside in the ganglion cell layer (GCL). Outer and inner plexiform layers (OPL and IPL, respectively) contain synaptic regions. (C) Schematic representation of the rod and cone photoreceptors, which have distinct compartmentalized morphology. The outer segment includes hundreds of membranous discs that contain visual pigment and other phototransduction components. The metabolic machinery is present in the inner segment. The visual proteins are transported to the outer segment via a connecting cilium. The nucleus is contained in the cell body, and the presynaptic region includes one or more ribbon-like structures for docking of synaptic vesicles.

During embryogenesis, the retina arises from neuroectoderm, which also generates other parts of the central nervous system. The retina is uniquely structured for perception, integration and transmission of visual information (Lamb et al., 2007). Six major types of neuron in the retina are organized in three cellular layers that are separated by synaptic layers (Fig. 1). Photoreceptors are the light-sensitive cells in the retina, with two distinct subtypes: rods and cones. Rod photoreceptors enable dim light vision, whereas cone photoreceptors mediate color vision and high visual acuity under brighter light conditions.

In the retina of most mammals, rods greatly outnumber cones, even in species that are largely diurnal; e.g. the human retina has ~105 million rods and 6 million cones. An additional cell layer, the RPE, underlies the retina and serves as a barrier between the photoreceptors and the choroidal blood supply. RPE plays crucial roles in supporting photoreceptor function, including two-way transport of nutrients and waste products and retinoid recycling (Fig. 1). Photoreceptors are highly specialized neurons designed for capturing light quanta and are organized in four distinct regions: the cell body, which includes the nucleus; the inner segment (IS); the outer segment (OS); and the synaptic region (Fig. 1C) (Lamb, 2013). The OS includes hundreds of stacked membranous discs carrying the proteins associated with phototransduction, including the visual pigment (opsin). The type of opsin present is unique to a photoreceptor subtype and defines its identity. Almost 10% of OS discs at the distal end are shed and phagocytosed by RPE daily, with new discs added at the proximal end, thereby renewing the complete OS in 10–15 days.

Three subtypes of cone photoreceptors can be generally identified in the human retina, based on the opsin they contain and its maximal spectral sensitivity; these are L- (long, 564 nm), M- (medium, 533 nm) and S- (short, 437 nm) wavelength cones. The mouse retina has only M- and S-cones. Only one type of rod photoreceptor, carrying the rhodopsin visual pigment, is present in the vertebrate retina, including in mouse and human. When in its ‘ready to be activated’ state, each opsin molecule is covalently bound to a light-sensitive chromophore, 11-*cis* retinal. Upon photon capture, the chromophore isomerizes to all-*trans* retinal, causing a conformational change in rhodopsin and activation to meta-rhodopsin II. This initiates the process of phototransduction, a cascade of biochemical events that culminate in closure of ionic channels in the cell membrane, hyperpolarization of the photoreceptor and transmission of the signal(s) to second-order neurons in the inner retina via modulation of neurotransmitter release at the synaptic terminals. All-*trans* retinal is then transported to the RPE for recycling and is returned to the photoreceptor in *cis* form, to allow production of new chromophore molecules (the visual cycle) (Travis et al., 2007).

The integrity and function of photoreceptors are absolutely crucial for vision, and mutations that affect photoreceptor function or survival disrupt the phototransduction process, leading to vision loss (Wright et al., 2010). In addition, defects in other retinal cell types, specifically the RPE, can also lead to photoreceptor dysfunction and retinal degeneration.

## Genetics of retinal degenerative diseases

Here, we briefly review RDDs before discussing relevant mouse models. A quick search of online Mendelian inheritance in man^®^ (OMIM; www.ncbi.nlm.nih.gov/omim/) shows over 1500 entries of inherited diseases with retinal dysfunction associated with over 200 different causative genes (RetNet), thus revealing tremendous clinical and genetic heterogeneity. RDDs exhibiting Mendelian inheritance can be subdivided into dominant, recessive and X-linked forms (Table 1) that can either solely impact retinal function or manifest as syndromic disease involving multiple tissues in addition to the retina. Interestingly, mutations in the same gene [e.g. *Peripherin* (also known as *RDS*), *CEP290*, *CRX*] can cause a range of clinical phenotypes (Boon et al., 2008; Coppieters et al., 2010; Sohocki et al., 1998), whereas similar phenotypes can be the end result of impairment in one of many different genes (RetNet). In other words, a clear one-to-one genotype-phenotype correlation is frequently not possible, and hereditary retinal degenerations are currently considered as probably the most genetically heterogeneous group of diseases in humans.

RDDs are usually classified into one of the two main clinical phenotypes – rod degenerative retinitis pigmentosa (RP) and cone or cone-rod dystrophy (CD or CRD, respectively) – that differ in the manner they affect rod versus cone photoreceptors. In RP, primary loss of rod photoreceptors occurs and is usually followed by cone dysfunction, whereas in ‘pure’ CDs the primary dysfunction or loss of cone photoreceptors might not necessarily cause secondary involvement of rods. When rods are involved in a primary CD, the disease is referred to as CRD. In early stages, the clinical phenotype usually reflects the primary cell type affected; i.e. in RP, night vision impairment often precedes subsequent visual field and visual acuity loss (owing to rod followed by subsequent cone impairment), whereas, in CD or CRD, the loss of visual acuity, impairment of color vision and photosensitivity (light aversion) are frequently the initial symptoms. In advanced RP and CRD, however, once widespread and severe retinal degeneration has developed, distinguishing between these two forms of disease can be difficult. In addition, it must be stressed that clinical phenotypes represent a wide spectrum, and the classification of these diseases is continuously being modified as molecular genetic insights are gained regarding the cause of disease. As mentioned above, in addition to primary mutations in rod- or cone-specific genes that might be associated with RDDs, mutations in genes associated with RPE function can also cause secondary photoreceptor disease because the RPE is crucial for photoreceptor homeostasis (Saari, 2012; Travis et al., 2007).

**Fig. 2.**
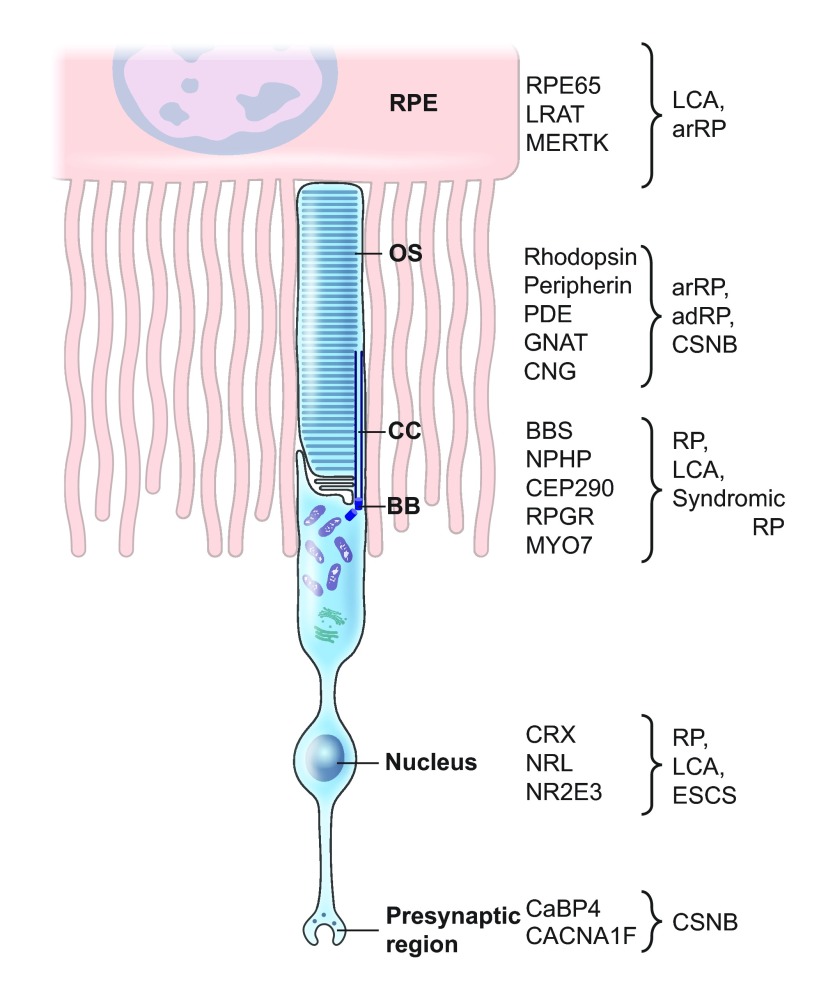
**A broad classification of proteins associated with retinal diseases according to their localization or function in photoreceptors and retinal pigment epithelium (RPE).** As illustrated, RPE65, LRAT and MERTK, which are associated with LCA and arRP, are RPE proteins, whereas CRX, NRL and NR2E3 are photoreceptor-specific transcription factors. The remaining disease-associated proteins that are listed localize to the outer segment (OS), connecting cilium (CC) and/or basal body (BB) of the photoreceptor (here a rod is represented). Abbreviations: adRP, autosomal dominant retinitis pigmentosa; arRP, autosomal recessive retinitis pigmentosa; CSNB, congenital stationary night blindness; ESCS, enhanced S-cone syndrome; LCA, Leber congenital amaurosis; RP, retinitis pigmentosa.

Fig. 2 illustrates the intimate relationship between photoreceptors and the RPE, and lists selected proteins, mutations in which cause RDDs, according to their specific localization. Among syndromic RDDs that involve other organs besides the retina, it is important to mention ciliopathies. Because the photoreceptor OS is a modified primary cilia (see Box 1), mutations in genes affecting cilia biogenesis or function often lead to retinal degeneration in addition to dysfunction of ciliated cells in other organs, such as the inner ear. Specific examples of syndromic ciliopathies with RDDs include Usher syndrome (in which varying degrees of hearing and vestibular function impairment occur in addition to retinal degeneration), Bardet-Biedl syndrome (BBS), Joubert syndrome and Senior-Loken syndrome (Table 1).

Macular degeneration (MD) is a specific form of RDD, affecting both rod and cone photoreceptors but limited to the macula, which is the central region of the human retina (Fig. 1A, top), responsible for high-resolution vision. The most common monogenic MD is Stargardt disease, a condition of early age onset, whereas AMD is a common and complex multifactorial disease with multiple genetic risk factors and onset in older age, as the name implies (recently reviewed in Fritsche et al., 2014).

## Animal models

The use of model organisms can facilitate the elucidation of cellular mechanisms underlying human disease. The fruit fly *Drosophila* is a classical model that has been used for defining fundamental pathways in vision, but its photoreceptor anatomy and physiology markedly differ from those of vertebrates. Zebrafish have become a model of choice for ocular developmental studies because of a closer phylogenetic link to humans and ease of genetic manipulations and experimentation (Avanesov and Malicki, 2010). Additionally, zebrafish embryos are transparent and can be obtained in large numbers. Among mammals, large animal models, particularly primates (that have a macula), might be better suited for understanding human disease; however, in addition to the ethical concerns involved, these animals are difficult to manage and manipulate genetically, are expensive to maintain, and only a limited number of spontaneously arising models of RDDs have been identified. Thus, rodent models, and particularly mice, have become the most widely used models of human disease. These small mammals are easy to manage in a laboratory environment, and multiple mouse mutants of retinal disease are already recognized or can be generated relatively easily for investigations. In addition, *in vivo* transfection or silencing of specific genes in mouse retina or *in vitro* transfection of retinal explants, using electroporation (Matsuda and Cepko, 2004) allows rapid examination of genes and variants. A list of naturally occurring and chemically induced mouse mutants as well as genetically engineered mouse models that manifest retinal disease is provided in Table 2 and supplementary material Table S1. This Review will focus on mouse mutants, which to date have been the primary animal models for exploring retinal disease pathogenesis and in designing novel treatments.

**Table 2.**
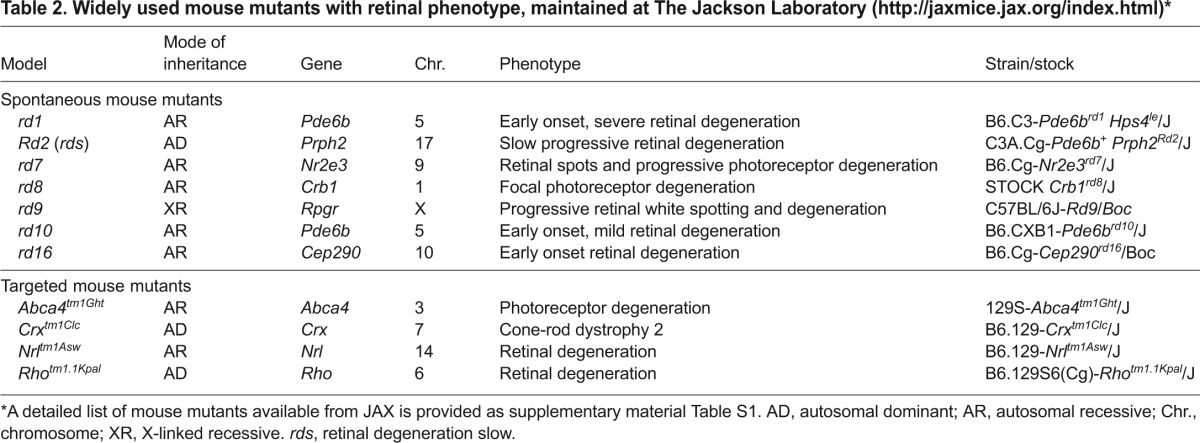
Widely used mouse mutants with retinal phenotype, maintained at The Jackson Laboratory (http://jaxmice.jax.org/index.html)*

## Evaluation of retinal degeneration phenotype

**Fig. 3.**
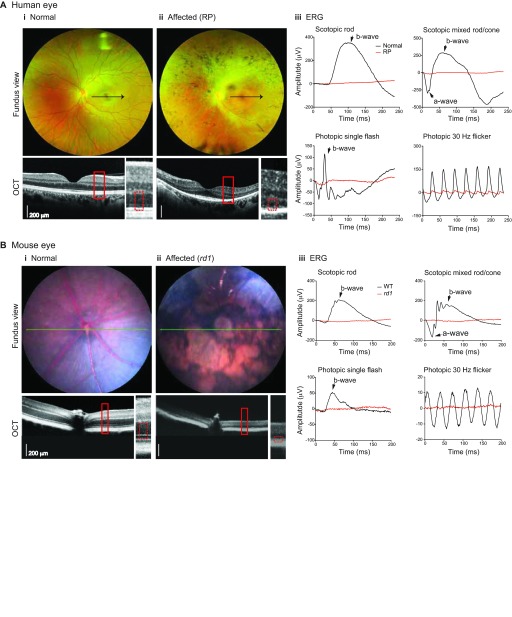
**Characterization of retinal degeneration in human patients and mouse mutants.** (A) Human ocular fundus photographs, optical coherence tomograms (OCT) and electroretinograms (ERG; see Box 1). (i) Wide-field color fundus image in an adult normal subject shows preserved macula and peripheral retina, with normal coloration of the underlying retinal pigment epithelium (RPE) and choroid. (ii) In an adult patient with retinitis pigmentosa [Affected (RP)], areas of atrophy accompanied by pigmentary changes indicate underlying photoreceptor degeneration. OCT imaging allows ‘histological-like’ assessment of retinal structure *in vivo*, including identification of different retinal layers. Whereas, in a normal subject, the photoreceptor layer (outer nuclear layer; see rectangular areas marked by the broken red line) is well preserved, marked thinning is evident in a patient with RP, with some sparing only in the area of the fovea, which contains only cone photoreceptors. This thinning reflects loss of photoreceptors as part of the progressive degeneration. The black arrow in the fundus images shows the location of the OCT scan across the macula, and the area in the red rectangle is magnified in the image to the right. (iii) ERG testing allows measurement of retinal function in response to light stimulation. Under dark-adapted conditions (scotopic), stimulation of the normal eye with a dim or bright white flash elicits a well-formed rod response (black traces, upper left panel) or mixed rod/cone response (upper right panel), respectively. In light-adapted conditions (photopic), single flash stimulation of the eye results in a normal cone response (lower left panel) whereas rapid stimulation (30 Hz) results in flicker waveform (lower right panel). By contrast, in RP patients, severe attenuation of these electrophysiological responses of the retina is evident (red traces). (B) Mouse ocular fundus photographs, OCT and ERG. (i) The normal [wild type (WT), C57BL/6J] mouse retina fundus has a uniform color and the blood vessels are visible. (ii) The *rd1* mutant mouse retina shows large areas of atrophy and discoloration, where the photoreceptors and possibly also the RPE have degenerated. The blood vessels are not visible in the degenerating retina. OCT imaging in a 3-month-old *rd1* mouse shows a striking difference in retinal thickness compared with the normal control (rectangular areas marked by broken red line). The OCT scan position is indicated by a green line in each fundus image. (iii) The dark-adapted (scotopic; indicating rod function) and light-adapted (photopic; indicating cone function) ERG responses are robust in the normal mouse (WT, black traces, at 3 weeks of age) and are practically non-detectable in the *rd1* mutant mouse (red traces). Anatomical and functional studies in mouse *rd* mutants are thus similar to what is generally observed in RP patients.

The eye and the retina, by virtue of their location, transparency, anatomy and physiology, allow detailed characterization of structure and function using an array of imaging, electrophysiological and psychophysical techniques that are largely non-invasive (Fig. 3). Examination of the ocular fundus (back of the eye visible through the pupil) by color photographs, fluorescein angiography, fundus autofluorescence and optical coherence tomography (OCT) imaging is routinely used to define retinal structure in health and disease in humans (Fig. 3Ai,ii) as well as in animal models (Fig. 3Bi,ii). Advanced techniques that correct for optical aberrations (adaptive optics) now allow imaging at the level of individual photoreceptors. Electrophysiological examinations, including electroretinography (ERG; see Box 1 for glossary) (Fig. 3Aiii–Biii) and electrooculography (EOG), permit quantification of retinal and RPE function, respectively. To a large extent, mouse models of retinal disease recapitulate the human disease, albeit at a different time course and with limitations that stem from the differences between the two species, such as life span and absence of a cone-rich macular region in mice.

In the following sections, we discuss different genes associated with RDDs that are grouped according to their primary role in retinal development and function. In each case, we provide reference to relevant mouse models and how they improved our understanding of disease pathogenesis, evolution and in some cases treatment. Similarities as well as differences between human disease and the mouse model are also addressed.

## RDDs affecting photoreceptor development

In photoreceptors, gene expression is under the stringent control of specific transcription factors, which include CRX (cone-rod homeobox), NRL (neural retina leucine zipper) and NR2E3 (nuclear receptor subfamily 2, group E, member 3) (Swaroop et al., 2010). Mutations of the genes encoding these transcription factors can globally affect photoreceptor development and homeostasis, leading to photoreceptor dysfunction and/or death. Some affected individuals are born blind and others develop blindness with age, depending on the gene and mutations involved. For example, mutations in *CRX* cause both early-onset Leber congenital amaurosis (LCA) and progressive CRD or RP, where disease progresses over time (Freund et al., 1997; Rivolta et al., 2001; Sohocki et al., 1998; Swaroop et al., 1999). Mutations in *NRL* and *NR2E3* cause RP with varying disease onset (Bessant et al., 1999; Haider et al., 2000; DeAngelis et al., 2002; Nakamura et al., 2004; Nishiguchi et al., 2004a; Wright et al., 2004). Mouse models have greatly augmented our understanding of photoreceptor cell fate determination and pathogenesis of retinopathies caused by mutations in transcription factors. Here we discuss CRX, NRL and NR2E3 in detail; however, mouse mutants have also established the role of OTX2, RORβ, TRβ2 and BLIMP1, among others, in photoreceptor development and disease (Brzezinski et al., 2010; Housset et al., 2013; Jia et al., 2009; Ng et al., 2001; Nishida et al., 2003; Roger et al., 2014).

CRX expression is largely restricted to photoreceptors in human and mouse retina (Chen et al., 1997; Freund et al., 1997; Furukawa et al., 1997), and it regulates the expression of numerous rod- and cone-specific genes (Corbo et al., 2010; Furukawa et al., 1999; Hao et al., 2012; Mitton et al., 2000). Mutations in *CRX* cause a spectrum of retinal disease phenotypes (Sohocki et al., 1998), including dominant CRD (Freund et al., 1997; Swain et al., 1997), RP and dominant as well as recessive LCA, in which marked retinal degeneration is already evident at birth (Rivolta et al., 2001; Swaroop et al., 1999). The key insight into CRX’s function in photoreceptors and its crucial role in photoreceptor development came from a mouse *Crx*-knockout (KO) model (Furukawa et al., 1999). *Crx*-KO mice are born blind, with non-functional photoreceptors that do not exhibit sufficient expression of many phototransduction genes, such as rhodopsin (*Rho*), compromising elaboration of rod outer segments (ROS) and ultimately resulting in photoreceptor degeneration. Surprisingly, whereas heterozygosity of specific *CRX* mutations in humans can cause severe retinal disease, *Crx^+/^*^−^ mice develop normal photoreceptors that do not degenerate (Furukawa et al., 1999). However, recently reported *Crx* mutants in mice can largely recapitulate the dominant LCA phenotype (Roger et al., 2014; Tran et al., 2014).

NRL is a retina-specific basic motif-leucine zipper (bZIP) transcription factor (Swaroop et al., 1992), which regulates the expression of hundreds of rod genes (Hao et al., 2012; Yoshida et al., 2004). In humans, mutations that affect NRL function by affecting its phosphorylation (Kanda et al., 2007) result in retinopathies (Bessant et al., 1999; Kanda et al., 2007; Nishiguchi et al., 2004a). Although NRL is only expressed in rods, heterozygous *NRL* mutations severely affect both rods and cones in affected individuals (DeAngelis et al., 2002). This is a common theme in RP; even when the genetic defect is in a rod-specific gene, cones eventually die for a variety of reasons, including the lack of trophic support. The *Nrl*-KO mouse was seminal in demonstrating that NRL is required for determination of rod fate (Mears et al., 2001) because the *Nrl*-KO retina has no rods and no expression of rod-specific genes. However, short-wavelength cones (S-cones) as well as S-opsin levels are markedly increased, and M-opsin levels are moderately enhanced. Notably, replacement of *Nrl* with thyroid hormone receptor (TR)-β2 in mice resulted in retina with M-cones instead of rods (Ng et al., 2011). This led to the hypothesis that S-cones are the ‘default’ fate, and expression of *Nrl* is required to switch on the molecular pathways that determine differentiation into the rod lineage (Swaroop et al., 2010), and that NRL and TR-β2 together determine different photoreceptor fates (Ng et al., 2011). Furthermore, global gene expression analysis of photoreceptors in the *Nrl*-KO mouse was instrumental in obtaining critical insights regarding genes and signaling pathways that are integral to rod homeostasis (Akimoto et al., 2006; Brooks et al., 2011; Yoshida et al., 2004; Yu et al., 2004). Ectopic expression of NRL in photoreceptor precursors produces only rods in mouse retina, implying its role as a master regulator in determining cone versus rod cell fate (Oh et al., 2007).

The significance of the *NR2E3* gene was recognized following the discovery that mutations in this gene cause enhanced S-cone syndrome (ESCS), with ‘gain-of-S-cone function’. The disease is progressive, often leading to marked visual impairment in later stages (Haider et al., 2000; Jacobson et al., 2004; Wright et al., 2004). In a post-mortem ESCS retina, the absence of rods and an excess of S-cones was confirmed (Milam et al., 2002). Elucidation of the function of NR2E3 and understanding of how mutations in this gene lead to ESCS came from analysis of a spontaneously arising mouse model, *rd7*, in which *NR2E3* is mutated and the human phenotype is recapitulated (Akhmedov et al., 2000; Cheng et al., 2011; Peng et al., 2005). NR2E3 is downstream of NRL (Oh et al., 2008) and *Nr2e3* expression is limited to post-mitotic rods (Bumsted O’Brien et al., 2004). In conclusion, NR2E3 has a dual role in reinforcing the rod cell fate while at the same time halting cone gene expression within the cell (Cheng et al., 2006).

In summary, the mouse models of *Crx*, *Nrl* and *Nr2e3* dysfunction were crucial in defining the mechanistic details of gene regulation in photoreceptors and establishing the basis for determination of photoreceptor fate during retinal development.

## RDDs caused by defects in intracellular trafficking and cilia function

The photoreceptor OS is a modified cilium, and the photoreceptor cell is elongated with the OS extending towards the underlying RPE. Therefore, proteins that are involved in ciliary development, function and intracellular trafficking are required for photoreceptor function. For example, trafficking of rhodopsin-carrying vesicles (possibly via microtubules) from the IS to the ROS is essential for OS morphogenesis and for phototransduction. Not surprisingly, mutations in genes required for development (e.g. the centrosomal *CEP290* gene) and maintenance of cilia or trafficking in cilia (e.g. Bardet-Biedl syndrome 4 homolog *BBS4* or the GTPase regulator-interacting protein *RPGR*) can lead to retinal dysfunction and degeneration (Rachel et al., 2012a).

LCA is a set of early-onset blinding diseases that are characterized by early and severe retinal dystrophy and low visual acuity practically from birth. LCA is associated with mutations in at least 19 genes (RetNet: https://sph.uth.edu/retnet/), with *CEP290* mutations accounting for almost 25% of the cases in North America. CEP290 is a centrosomal-cilia protein (Chang et al., 2006b) that is highly expressed in neural retina and nasal epithelium of humans (Papon et al., 2010). Mutations in *CEP290* are also associated with Joubert syndrome (Sayer et al., 2006; Valente et al., 2006), Senior-Loken syndrome (Sayer et al., 2006), BBS (Leitch et al., 2008) and Meckel syndrome (Baala et al., 2007), in addition to LCA (Cideciyan et al., 2007; den Hollander et al., 2006). Insights into disease pathology associated with *CEP290* mutations came from a spontaneous mouse mutant, *Cep290^rd16^* (Chang et al., 2006b; Cideciyan et al., 2011). The *Cep290^rd16^* mouse displays early-onset retinal degeneration with mis-localization of RPGR and rhodopsin in the photoreceptors (Chang et al., 2006b). The retinal phenotype in *Cep290^rd16^* was intriguingly rescued in the McKusick-Kaufman syndrome 6 (*Mkks6*) mutant background; however, the mechanism of rescue is not understood (Rachel et al., 2012b).

Almost 70% of X-linked retinitis pigmentosa (XLRP; RP3) can be accounted for by mutations in the *RPGR* gene (Breuer et al., 2002; Vervoort et al., 2000; Zito et al., 2000). *RPGR* mutations are also detected in RP patients (specifically males) where no family history is available, in apparently autosomal dominant RP families, and in patients with CRD and MD (Ayyagari et al., 2002; Branham et al., 2012; Churchill et al., 2013; Demirci et al., 2002; Sharon et al., 2003). Studies with the *Rpgr*-KO mouse permitted the investigators to suggest its possible role in connecting cilia and directional transport needed for photoreceptor survival (Hong et al., 2003; Hong et al., 2000). The *Rd9* mouse was identified as a naturally occurring mutant mouse that lacks the functional RPGR protein due to a frameshift mutation within the region of open reading frame 15 (ORF15) (Thompson et al., 2012). These two and another conditional knockout mouse model have been valuable for designing gene therapy vectors for treatment of human disease caused by *RPGR* mutations (Hong et al., 2005; Huang et al., 2012).

BBS is another genetically heterogeneous syndromic ciliopathy, with high incidence of retinal dystrophy together with polydactyly, urinary system abnormalities, obesity, renal failure, varying degrees of mental retardation and cardiovascular complications. BBS is associated with mutations in at least 19 genes. We provide three examples here. *BBS2* and *BBS4* mutations cause BBS (Mykytyn et al., 2001; Nishimura et al., 2001), whereas *BBS6* (or *MKKS*) gene defects are associated with McKusick-Kaufman syndrome (abnormalities in finger, heart and genitals) in addition to BBS (Katsanis et al., 2000; Slavotinek et al., 2000). BBS2 and BBS4 proteins are constituents of the BBSome, a component of the basal body that is involved in formation of the nonmotile primary cilium (see Box 1) (Kim et al., 2004; Nachury et al., 2007; Shah et al., 2008), and BBS6 is a component of the chaperonin complex (see Box 1) that is required for assembling the BBSome (Seo et al., 2010). The analysis of KO mice has revealed that BBS2 and BBS4 are required for photoreceptor maintenance. In *Bbs4*-KO mice, the photoreceptors degenerated much earlier than in *Bbs2*-KO mice (Mykytyn et al., 2004). BBS4 is also required for formation of spermatozoa flagella, but is not required for nonmotile primary cilia in other tissues (Nishimura et al., 2004). These mice recapitulated some but not all aspects of the human syndrome (Mykytyn et al., 2004). The photoreceptor degeneration is comparable in *Bbs2*-KO, *Bbs4*-KO and *Bbs6*-KO mice (Fath et al., 2005; Ross et al., 2005). In summary, the studies in rodent models have suggested that BBS genes are required for the maintenance of cilia function in photoreceptors, and mutations in BBS genes likely compromise trafficking of proteins to the cilium. Rodent models are not available for many BBS genes, and creation of additional models would facilitate the functional analysis of their role in cilia.

Usher syndrome is another genetically heterogeneous group of disorders that are characterized by RP along with congenital or progressive sensory deafness and varying degrees of vestibular dysfunction. Type I Usher syndrome, the most severe form, is mostly caused by mutations in *MYO7A* (*myosin VIIA*; also known as *USH1B*) (Le Quesne Stabej et al., 2012; Weil et al., 1995). *MYO7A* encodes an unconventional myosin motor protein and is expressed in human embryonic RPE, photoreceptors, cochlear and vestibular neural epithelia (Weil et al., 1996). The shaker mouse (*sh1*) is a naturally occurring model with a spontaneous mutation in *Myo7a*, and manifests deafness and vestibular dysfunction. The photoreceptors in *sh1* mice have accumulation of opsin at the base of the cilium, suggesting a role for MYO7A in opsin transport (Liu et al., 1999). A second Usher gene, *USH2A* (Usher syndrome 2A) encodes an enormously large matrix protein and is expressed in the photoreceptors and cochlear hair cells. Mutations in *USH2A* are associated with type II Usher syndrome (Eudy et al., 1998), in which RP is invariably present but the hearing deficit is of later onset, progressive and variable. Indeed, some mutations in *USH2A* result only in RP (*RP39*) without involvement of the inner ear (Rivolta et al., 2002). Mutations in *USH2A* are a common cause of autosomal recessive RP (arRP), accounting for 10–15% of cases. The analysis of *Ush2a*-KO mice revealed that Usherin, the protein encoded by this gene, wraps around the connecting cilia at the boundary of inner and outer segments and is required for the maintenance of photoreceptors (Liu et al., 2007). After the development of the structural components, additional proteins such as Usherin seem to be required for functional integrity of the photoreceptors. In contrast, Usherin is required for the development of cochlear hair cells (Liu et al., 2007). We note that mouse models of Usher syndrome generally do not exhibit significant photoreceptor disease (Gibson et al., 1995).

The molecular carriers required for trafficking of rhodopsin to the ROS are not yet fully understood. RAB proteins are small GTPases involved in subcellular trafficking of membranes and have been suggested to mediate rhodopsin trafficking (Deretic, 1997). Rab3A, Rab6, Rab8 and Rab11 have been implicated in trafficking of rhodopsin from the sorting organelle known as the Golgi apparatus to the connecting cilium (Deretic et al., 1996; Mazelova et al., 2009). However, the analysis of *rab8a* and *rab8b* double-KO mice ruled out their requirement for ciliogenesis and/or photoreceptor development (Sato et al., 2014). No retinal diseases associated with RABs have been identified, except for CRD, which likely arises from a mutation in *RAB28* (Roosing et al., 2013). The localization of RAB28 to the basal body and ciliary rootlet suggests a role in ciliary transport. Mouse mutants of Rab28 would facilitate functional analysis of Rab28 in intracellular transport and provide insights into disease mechanism.

Tulp1 (tubby like protein 1) is expressed in the retina and is implicated in trafficking of rhodopsin (Hagstrom et al., 1999; Ikeda et al., 2000). Mutations in *TULP1* are associated with arRP (Banerjee et al., 1998; Hagstrom et al., 1998) and LCA (Hanein et al., 2004). The *Tulp1*-KO mouse indeed manifests early-onset retinal degeneration with rapidly progressive loss of photoreceptors.

The structural scaffold for membranous discs in the ROS is dependent on peripherin (RDS) and its interactor protein ROM1 (retinal outer segment membrane protein 1). Peripherin is localized to the rim of the OS in rods and cones, and is essential for their formation and renewal (Connell et al., 1991; Travis et al., 1991). Not surprisingly, mutations in *peripherin2* (*PRPH2*) are associated with a variety of retinal degeneration phenotypes in humans, such as CRD and autosomal dominant RP (adRP) (Keen and Inglehearn, 1996; Nakazawa et al., 1996). The ‘retinal degeneration slow’ (*rds*) mouse (*Rd2*) is the classical model for retinal degeneration (Sanyal and Bal, 1973) and carries a *Prph2* mutation (Travis et al., 1991). Homozygous *rds* mice are unable to elaborate the OS, and photoreceptors degeneration begins as early as postnatal day 14, but this loss progresses relatively slowly over the span of 1 year (Sharma et al., 2012). Following identification of human RP patients who were doubly heterozygous for a mutation in *PRPH2* (*RDS*) and a null mutation in *ROM1* (Dryja et al., 1997; Kajiwara et al., 1994), a similar digenic mutant mouse model showed faster photoreceptor degeneration compared with *rds* mutation alone, and a positive correlation was observed between the rate of photoreceptor loss and the extent of OS disorganization (Kedzierski et al., 2001).

## RDDs caused by phototransduction defects

**Fig. 4.**
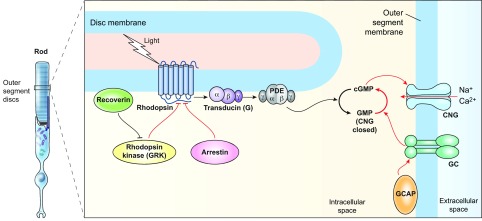
**Schematic view of major proteins involved in phototransduction.** The phototransduction events are broadly similar in rod and cone photoreceptors, and, given their complexity, we show here only the key proteins associated with rod phototransduction. During phototransduction (black arrows), the capture of photon(s) results in activation of rhodopsin, leading to dissociation of transducin (G protein) subunits βγ from Gα, which in turn activates cGMP-phosphodiesterase (PDE). PDE catalyzes the hydrolysis of cGMP to GMP, thereby causing closure of cyclic-nucleotide-gated (CNG) channels in the photoreceptor outer segment membrane. The closure of CNG channels results in photoreceptor hyperpolarization and transmission of the electrochemical signal(s) to second-order neurons in the inner retina via modulation of neurotransmitter release (not shown here). Channel closure also blocks Ca^2+^ entry, resulting in reduced intracellular Ca^2+^ (not shown here) and transmission of a feedback signal for recovery by engaging guanylyl cyclase activating proteins (GCAP). At low Ca^2+^ levels, GCAP activates guanylate cyclase (GC) and stimulates cGMP synthesis, thereby restoring cGMP levels and leading to re-opening of CNG channels. Termination of phototransduction (red arrows and T bars) also requires the inactivation of rhodopsin, which is initiated by its phosphorylation by rhodopsin kinase [G-protein receptor kinase (GRK)], facilitating the binding of arrestin to rhodopsin. In the dark and at high intracellular Ca^2+^ levels, recoverin inhibits GRK and controls the life time of activated rhodopsin. The transducin-PDE complex is inactivated by the hydrolysis of bound GTP that is greatly accelerated by the RGS9 complex (not shown here). The latter consists of regulators of G protein signaling member 9 (RGS9), G protein β5 and RGS9 associated protein (R9AP) (not illustrated in the figure). The negative feedback loop associated with Ca^2+^ concentration is critical for maintaining phototransduction.

Visual transduction is initiated by a cascade of biochemical reactions (Fig. 4), and mutations in genes encoding phototransduction proteins are associated with blindness. Mutations in rhodopsin and cone opsins, which initiate phototransduction in rod and cone photoreceptors, respectively, can cause photoreceptor dysfunction with or without degeneration. In the rods, for example, G90D, T94I, A292E and A295V rhodopsin mutations result in a form of congenital night blindness (nyctalopia) despite rod photoreceptors being maintained nearly intact across the human life span (Dryja et al., 1993; Sieving et al., 1995; Zeitz et al., 2008). By comparison, many rhodopsin mutations (such as T17M, P23H) are associated with night-blindness from a degenerative progressive RP phenotype. K296E and K296M rhodopsin mutations cause adRP with early severe photoreceptor degeneration and vision loss (Keen et al., 1991; Vaithinathan et al., 1994). Interestingly, some of these mutations result in constitutive activity of opsin (Rao et al., 1994; Robinson et al., 1994; Zeitz et al., 2008); however, the disease phenotypes are profoundly different, suggesting that the mechanism of disease is distinct in many cases. For example, analysis of the G90D mutation using a transgenic mouse revealed sufficient activity of the chromophore-free opsin that interferes with the ability to perceive dim, real environmental light against the background of intrinsic spurious light, thereby causing vision loss at night (Sieving et al., 2001).

Rhodopsin is a major structural protein of the ROS. In patients with T17M and P23H rhodopsin mutations, which are a common cause of adRP (Hartong et al., 2006), rhodopsin is not targeted to the outer segments, resulting in short ROS (Li et al., 1994). A transgenic mouse model with humanized rhodopsin carrying the P23H mutation confirmed defective rhodopsin transport (Olsson et al., 1992). P23H is inherently unstable and its regeneration is slower compared with wild-type rhodopsin (Chen et al., 2014). Recently, the analysis of P23H knock-in mice revealed a new step in OS disc biogenesis (Sakami et al., 2014), explaining the cause of structural defects in discs and consequently photoreceptor degeneration. The *Rho*-KO mouse model demonstrated that a single copy of *Rho* is sufficient to drive both development and function of the ROS in mice, yet both alleles are required to maintain long-term functional integrity of the photoreceptors (Humphries et al., 1997; Lem et al., 1999).

Termination of phototransduction requires deactivation of activated rhodopsin (R*) and of transducin-PDE (G*-PDE*), and mutations in genes associated with this process cause retinal dysfunction and degeneration. Light-dependent deactivation of rhodopsin is accomplished in a two-step process. First, rhodopsin is phosphorylated by rhodopsin kinase (GRK1) followed by arrestin binding to rhodopsin. Mutations in *GRK1* are associated with Oguchi disease-2 (CSNBO2), which results in delayed dark adaptation (see Box 1) (Cideciyan et al., 1998; Yamamoto et al., 1997). Analysis of *Grk1*-KO mice demonstrated that GRK1-mediated light-dependent phosphorylation is required for deactivation of activated rhodopsin, and absence of GRK1 leads to photosensitization of the rods and induces apoptotic death (Chen et al., 1999). Mutations in *SAG*, which is required for terminating rhodopsin activation, are primarily associated with Oguchi disease-1 (Maw et al., 1998; Nakamura et al., 2004; Waheed et al., 2012), but some mutations can cause an RP phenotype (Nakazawa et al., 1998). Studies with knockout mice revealed that arrestin does not initiate but completes the quenching of rhodopsin’s catalytic activity (Xu et al., 1997). Mouse models have also permitted the identification of fundamental differences in the presence and function of arrestin proteins in rod and cone photoreceptors as well as in different species; e.g. cone arrestin that is ectopically expressed in rods binds less efficiently to phosphorylated rhodopsin, compared with rod arrestin (Chan et al., 2007; Nikonov et al., 2008; Weiss et al., 2001).

In rods, the α-subunit of the heterotrimeric G protein transducin is encoded by *GNAT1* (Lerman and Minna, 2000), and patients with missense mutations in *GNAT1* exhibit congenital stationary night blindness (CSNB) (Dryja et al., 1996; Naeem et al., 2012). A missense mutation in *GNAT1* leading to a G38D change in the protein causes Nougaret CSNB, with ~100-fold reduction in rod sensitivity (Dryja et al., 1996). Mimicking this mutation in a transgenic mouse revealed reduced GTPase activity of GNAT1 and its ability to activate PDE6 (Moussaif et al., 2006). However, in contrast to affected humans, mice with the G38D mutation in the heterozygous state do not display reduced rod sensitivity. The *Gnat1*-KO mouse demonstrated that rod-driven signals require functional GNAT1, and its absence leads to slow degeneration of the photoreceptors (Calvert et al., 2000). *GNAT*2 encodes the cone version of α-transducin (Morris and Fong, 1993), and mutations in *GNAT2* result in complete achromatopsia – i.e. no cone function at all (Kohl et al., 2002) – incomplete achromatopsia or extreme red-green color blindness (protanopia) (Rosenberg et al., 2004). The *Gnat2*-KO mouse phenotype largely resembled the human disease and revealed that misfolding of the transducin protein results in loss of cone function, opsin mis-localization, retinal remodeling and slow degeneration of photoreceptors (Jobling et al., 2013). Largely similar findings were obtained in another spontaneously occurring model, *Gnat2^cpfl3^* (Chang et al., 2006a), which demonstrated the efficacy of gene augmentation therapy using an AAV5 vector carrying a normal mouse *Gnat2* gene under control of the red-green opsin promoter (Alexander et al., 2007).

The heterotetrameric PDE6 complex regulates intracellular cGMP levels by hydrolyzing cGMP in response to light activation and is thus a key component in the phototransduction cascade (see Fig. 4). Null or missense mutations in *PDE6A*, which encodes the α-subunit of this protein, are associated with arRP (Dryja et al., 1999; Petersen-Jones et al., 1999). Rapid photoreceptor degeneration is detected in a mouse model carrying a *Pde6a* missense mutation, without the induction of apoptosis (Sakamoto et al., 2009). This mutation affects the catalytic domain of PDE6A, required for maintaining PDE6B levels in the retina. Missense or truncating mutations toward the C-terminus of PDE6B (encoding the β-subunit) also result in arRP (McLaughlin et al., 1993). The naturally occurring *Pde6b^rd1/rd1^* (*rd1*) mouse (Sidman and Green, 1965) develops rapid photoreceptor degeneration (Caley et al., 1972; Sanyal and Bal, 1973), whereas another mutation in this gene in the *Pde6b^rd10^* mouse displays a somewhat milder phenotype (Gargini et al., 2007). The *rd10* mouse model is often used for testing therapeutic interventions of RP (Chang et al., 2002). In humans, the H258N mutation in *PDE6B* results in an autosomal dominant CSNB phenotype (Gal et al., 1994). An attempt to recapitulate the human phenotype by expressing the *H258N* transgene in mice did not succeed (Tsang et al., 2007), but a single allele of H258N *Pde6b* rescued the photoreceptor degeneration in *Pde6b^rd1/rd1^* mice (Farber and Lolley, 1976).

The PDE heterotetramer also contains two γ-subunits, encoded by the *PDE6G* gene. Only one large consanguineous family manifesting an early-onset RP phenotype has been reported to have a *PDE6G* mutation (Dvir et al., 2010). Analysis of transgenic *Pde6g^tm1^* mice revealed that cGMP levels were initially increased in photoreceptors (Tsang et al., 1996). Analysis of *Pde6g* mutant mice (*Del7C* transgenic) showed that the PDE6G C-terminus has no independent catalytic function because it could not rescue *Pde6g^tm1^* mice (Farber and Tsang, 2003). On the other hand, *Pde6g* transgenic mice with a Y84G mutation rescues *Pde6g^tm1^* mice (Tsang et al., 2001). Similarly, a W70A mutant *Pde6g* transgene, thought to affect the affinity of PDE6G for transducin, rescues *Pde6g^tm1^* mutant mice, but α-transducin GTPase hydrolysis was slower. In W70A *Pde6g* transgenic mice, a model of stationary nyctalopia, the rods are highly insensitive to light (Salchow et al., 1999).

The PDE complex also contains PDE-delta protein, and mutations in the PDE-delta gene are associated with Joubert syndrome (Barker et al., 2014). The PDE-delta-KO mouse displays recessive CRD (Zhang et al., 2007), and this model allowed researchers to identify the role of PDE-delta in trafficking of lapidated proteins such as GRK12 and PDE6 in the photoreceptor.

Several proteins in the phototransduction cascade have a farnesyl group added for membrane attachment. *AIPL1* (aryl-hydrocarbon-interacting protein-like 1) mutations are associated with LCA4, a severe early-onset retinal degeneration (Sohocki et al., 2000). In *Aipl1*-KO mice, PDE farnesylation is undetectable and rod cGMP levels are elevated, leading to apoptotic death of rods and subsequently cones (Ramamurthy et al., 2004). The rod but not cone degeneration is rescued by the human ortholog of *AIPL1* (Kirschman et al., 2010), suggesting that AIPL1 function is restricted to rods (van der Spuy et al., 2002). AIPL1 interacts with the α-subunit of PDE6 and is essential for assembly of PDE6 subunits (Kolandaivelu et al., 2009).

In rods, the inward negative current flowing in the dark-adapted state (the so-called ‘dark current’) is a result of sodium and calcium influx through open cyclic-nucleotide-gated (CNG) channels. Light initiates the phototransduction cascade, resulting in the closure of CNG channels, which generates a hyperpolarization wave in the photoreceptors. CNG channels comprise α- (CNGA) and β- (CNGB) subunits (Kaupp and Seifert, 2002). Mutations in *CNGA1* and *CNGB1* cause arRP (Bareil et al., 2001; Dryja et al., 1995). A *Cngb1*-KO mouse displayed rod degeneration (Hüttl et al., 2005). Although, initially, cone function was preserved, by 1 year of age both cones and rods were lost. In the absence of CNGB1, the level of CNGA1 was also reduced in the OS, suggesting that the CNGB1 subunit is required for proper targeting of the CNGA1 subunit. The *Cngb1* locus also encodes two related glutamic-acid-rich proteins (GARPs) (Körschen et al., 1995), and deletion of CNGB1 along with the GARPs in the null *Cngb1-X1* mouse markedly affected photoreceptor disk morphogenesis (Zhang et al., 2009). Mutations in *CNGA3* and *CNGB3*, which encode similar subunits in cone photoreceptor channels, are a major cause of achromatopsia (Kaupp and Seifert, 2002; Kohl et al., 1998). Mouse models of CNGA3 and CNGB3 achromatopsia showed cone dysfunction and have been used to evaluate the efficacy of gene augmentation therapy (Biel et al., 1999; Carvalho et al., 2011; Pang et al., 2012). A *Cngb3*-KO mouse (as well as additional mouse models of cone disease) was also recently used to examine the effects of thyroid hormone on cone survival, as a potential novel therapeutic approach (Ma et al., 2014).

The rate-limiting step in the termination of phototransduction is the deactivation of activated transducin-PDE (G*-PDE*) (Krispel et al., 2006). This is accomplished by two important steps: GTPase activating protein [GAP; consisting of ‘regulators of G protein family member 9’ (RGS9), RGS9 associated protein (R9AP) and G protein β5] deactivates the G*-PDE* complex, and guanylate cyclases (GCs) with their activators (GCAPs) replenish cGMP. Whereas *R9AP* and *RGS9* mutations cause bradyopsia (see Box 1) (Nishiguchi et al., 2004b), *GC1* mutations result in CD and LCA (Hanein et al., 2004). On the other hand, *GCAP1* mutations have been associated with CRD (Baehr et al., 2007). In mice, GC1 is expressed both in rods and cones, whereas GC2 is expressed only in rods; these two GCs maintain the dark current in rods and their function seems redundant or overlapping, as revealed by *GC1*-KO or *GC2*-KO mice. Notably, the double KO has nonfunctional rods and cones (Baehr et al., 2007). The analysis of a GCAP1 and GCAP2 double-knockout mouse reveals a photoresponse with larger amplitude and delayed decline compared with the wild type (Mendez et al., 2001).

Given the importance and complexity of the visual process in mammals, it is not surprising that mutations in almost all proteins that are associated with photoreceptor function, specifically phototransduction, can cause vision impairment. Here, model organisms (particularly mouse mutants) have been invaluable in elucidating disease mechanisms and designing of treatments (discussed later).

## RDDs and synaptic transmission defects in mouse models

Photons captured by photoreceptors are transduced into an electrochemical signal at the ribbon synapses with bipolar cells. The vesicles carrying glutamate neurotransmitter mediate this step by releasing their content in response to changes in the membrane potential. In the dark, photoreceptor L-type calcium channels are open and Ca^2+^ influx to the cytoplasm occurs, causing glutamate release. Following activation by light, a graded decrease in glutamate release at the ribbon synapses mediates the signal onwards from the photoreceptor to the bipolar cell. The main Ca^2+^ channel in rod and cone synapses is the calcium-binding protein CaBP4 (Haeseleer et al., 2004). *CACNA1F* encodes a subunit of the voltage-gated L-type calcium channels expressed in the retina, and mutations in this gene cause X-linked CSNB (Strom et al., 1998) and, less frequently, X-linked CRD (Huang et al., 2013; Jalkanen et al., 2006). This might be due to an abnormal Ca^2+^ influx and neurotransmitter release that compromises membrane potential at the outer plexiform layer (OPL; Fig. 1B) (Ball et al., 2002; Haeseleer et al., 2004). *Cacna1f*-KO mice show reduced rod and cone ERG and loss of photoreceptor synapses (Mansergh et al., 2005). Mutations in *CACNA2D4*, another subunit of the voltage-gated L-type calcium channel, are associated with autosomal recessive CRD (arCRD) (Wycisk et al., 2006). Furthermore, a naturally occurring *Cacna2d4* mutant mouse displayed retinal degeneration with marked defects in the synaptic layer.

Unlike conventional glutamate synapses, the photoreceptor ribbon synapses are not dependent on proteins of the Munc13 family for exocytosis (Cooper et al., 2012). This fundamental difference of the ribbon synapse was identified through analysis of mice lacking the ubiquitously expressed Munc13-2 splice variant (*ubMunc13-2*-KO mice), indicating specialized machinery to mediate exocytosis in ribbon synapses. Although the molecular components in this process are still largely unknown, the significance of one key component, CSP-α (DNAJC5), in vision has been demonstrated using null mice (Schmitz et al., 2006). Lack of CSP-α in photoreceptor terminals impaired assembly of the SNARE complex, which is required for membrane fusion and the development of ribbon synapses, leading to progressive neurodegeneration (Sharma et al., 2011).

UNC119 (uncoordinated 119) is expressed in ribbon synapses of rods and cones, and a mutation in *UNC119* was linked to late-onset CRD in one patient. However, its involvement in CRD is debatable, because the mutation did not co-segregate with the disease in the family (Kobayashi et al., 2000). Transgenic mice carrying a mutated human *UNC119* transgene develop fundus lesions, display abnormalities in ribbon synapses and abnormal ERG responses, suggesting that retinal degeneration might be caused by defects in trans-synaptic transmission (Kobayashi et al., 2000). Further investigations are however required.

RIMS1 (regulating synaptic membrane exocytosis 1) is a RAB3A-interacting protein (Wang et al., 2000), and RAB3A is a synaptic-vesicle-associated protein involved in exocytosis. Mutations in *RIMS1* cause autosomal dominant CRD (adCRD) (Johnson et al., 2003), and the *Rims1*-KO mouse indeed shows defects in neurotransmitter release (Schoch et al., 2002).

Currently, we have limited knowledge of the molecular determinants at retinal synapses. In the future, forward- and reverse-genetic approaches using mouse models could be adopted to identify new genes and pathways affecting the structure and function of photoreceptor and other synapses in the retina.

## RDDs caused by defects in RPE integrity or function

We now discuss defects in the RPE, which plays a crucial role in photoreceptor survival. Mutations in several RPE-specific genes, including *RPE65*, *LRAT* (lecithin retinol acyltransferase) and *MERTK* (tyrosine-protein kinase Mer), have been identified in patients with early-onset retinal degeneration and LCA (Gal et al., 2000; Gu et al., 1997; Thompson et al., 2001). Currently, three mouse models are available for investigating *RPE65*, which encodes an isomerohydrolase that is crucial for the derivation of *cis*-retinal: first, a naturally occurring mouse mutant, *rd12*; second, an *Rpe65*-KO model; and, finally, a transgenic mouse carrying the R91W mutation that is often seen in humans (Pang et al., 2006; Redmond et al., 1998; Samardzija et al., 2008). The null and *rd12* models display degeneration of photoreceptors, but the *Rpe65*-KO model has a preponderance of loss of S-cones. *RPE65-R91W* transgenic mice are able to generate some 11-*cis*-retinal (~10% of normal), which leads to partially functional rhodopsin, and the rate of degeneration in this mutant is somewhat slower than in the *Rpe65*-KO mouse. Differences in mouse mutants might reflect observed phenotypes among patients with distinct *RPE65* mutations (Cideciyan, 2010) (R. Ratnapriya, E.B., S. G. Jacobson and A.S., unpublished data). Analysis of *Lrat*-KO mice has demonstrated a requirement of LRAT in RPE for ROS maintenance (Batten et al., 2004): ERG recordings were severely reduced in *Lrat*-KO mice at a young age. Thus, this mutant serves as a good model for early-onset severe retinal dystrophy such as that occurring in LCA. The functional role of MERTK was initially analyzed in the Royal College of Surgeon (RCS) rat and then in *Mertk*-KO mice, which demonstrate retention of discarded disc material between the photoreceptors and the RPE, resulting in gradual loss of photoreceptors (Duncan et al., 2003).

## Other degenerative diseases

### Stargardt disease

Stargardt disease is the most common form of genetically driven progressive juvenile macular degeneration that affects central vision. In the original use of the term, Stargardt is inherited in an autosomal recessive mode from mutations in the gene *ABCA4* [ATP-binding cassette, sub-family A (ABC1), member 4], which encodes an ATP-binding transporter protein (Allikmets, 1997) that is specifically expressed in photoreceptors. ABCA4 functions as a flippase, which moves N-retinylidene-phosphatidylethanolamine (NR-PE) from inside the ROS to the outside. The *Abca4*-KO mouse model has been utilized to understand the etiology of Stargardt disease (Weng et al., 1999). In *Abca4*-KO mice, phagocytosis of the ROS by the RPE results in the accumulation of A2-E (N-retinylidene-N-retinylethanolamine) to toxic levels in the RPE.

Some cases of Stargardt disease follow autosomal dominant inheritance owing to mutations in *ELOVL4* (elongation of very long chain fatty acids-like 4) (Allikmets, 1997; Zhang et al., 2001), which is required for the synthesis of very-long-chain fatty acids (Agbaga et al., 2008). Analysis of humanized transgenic mice expressing mutant *ELOVL4* reveals that RPE atrophy and photoreceptor degeneration result from accumulation of phagosomes and lipofuscin (Karan et al., 2005; Vasireddy et al., 2006).

### Retinoschisis

X-linked retinoschisis (XLRS) is a prevalent retinal dystrophy affecting only males and marked by the schisis (splitting) of the neural retina. XLRS is caused by mutations in the retinoschisin gene (*RS1*) (Hiriyanna et al., 1999; Sauer et al., 1997). Retinoschisin is a cell-surface adhesion molecule expressed by photoreceptor and bipolar cells, and is required for the development and maintenance of retinal architecture (Vijayasarathy et al., 2007). Analysis of *Rs1*-KO mice showed pan-retinal pathological splitting of the retina (Weber et al., 2002), which mimics human XLRS1 disease (Prenner et al., 2006). One difference in humans is the distinctive radiating pattern of macular schisis cysts, which are not replicated in mouse owing to the lack of a macular structure. The findings in *Rs1*-KO mice support the notion that this protein is required for the organization of retinal layers and for organization and function of the photoreceptor-bipolar cell synapse, thereby explaining the characteristic reduction of the ERG b-wave, which is generated by the bipolar cells following trans-synaptic activation by the photoreceptors. Furthermore, in *Rs1*-KO mice the a-wave (produced by the hyperpolarization of photoreceptors) is preserved, supporting normal initiation of the visual signal (Takada et al., 2008). Importantly, *Rs1*-KO mouse models have served to show the possibility of gene therapy via intravitreal delivery of viral vectors carrying the normal gene, in preparation for application of this treatment in patients with retinoschisis (Byrne et al., 2014; Park et al., 2009; Zeng et al., 2004).

### Leber hereditary optic atrophy

Leber hereditary optic atrophy (LHOA) often manifests in the second decade of life and is the cause of acute or subacute central vision loss. LHOA results from mutations in mitochondrial genes; some of the mutations can also induce neurological and muscular phenotypes (Larsson et al., 1991). Many alleles are associated with LHOA, but three primary mutations (at nucleotide positions −3460, −11778 and −14484 of LHOA, affecting Complex I) underlie the majority of cases (Riordan-Eva and Harding, 1995). Given its multigenic and complex inheritance pattern, designing an animal model has been quite challenging. The animal models for LHOA have been generated by reducing *SOD2* mRNA levels in the eye (Qi et al., 2003) or by delivering mutant versions of the human *ND4* (NADH dehydrogenase subunit 4) gene into the eye (Ellouze et al., 2008; Qi et al., 2007). Such localized alteration of gene expression in the eye replicated clinical features of LHOA, with disrupted mitochondrial cytoarchitecture and death of the ganglion cells (Qi et al., 2007). The use of wild-type human *ND4* has been promoted as a safe option for treating LHOA because delivery of *ND4* to the mouse eye did not elicit side effects (Ellouze et al., 2008). An induced mouse model has also been used in preclinical studies of *ND4* gene therapy for LHOA (Koilkonda et al., 2014). A human clinical trial was started in 2014 using adeno-associated virus 2 (AAV2) delivery to target the mitochondrial *ND4* gene mutation, G11778A (www.clinicaltrials.gov NCT02161380) (Lam et al., 2014).

## Preclinical models for developing therapies

**Fig. 5.**
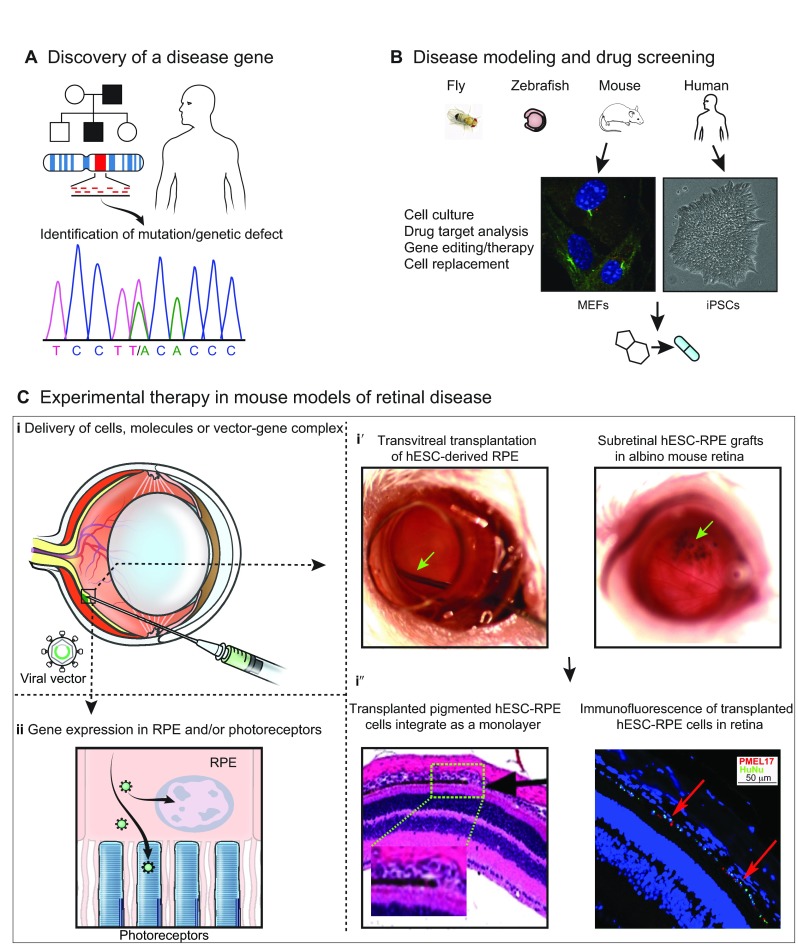
**From gene discovery to therapy of retinal degenerative diseases.** (A) Schematic representation of the discovery of a gene associated with retinal disease. To identify the genetic defect associated with a diseases phenotype, DNA sequences of affected individuals (black squares) are compared with those of healthy individuals (white squares and circles) in the family. A hypothetical genetic difference in an individual with dominant disease is shown in the sequence (T/A). (B) Paradigms for retinal disease modeling and drug discovery. *Drosophila* (fly) and zebrafish embryos are useful for high-throughput large-scale drug screening; mouse models are excellent for elucidating disease mechanisms and for testing therapies; small-molecule (drug) screenings are often performed using cell culture systems derived from either mouse embryonic fibroblasts (MEFs) or human induced pluripotent stem cells (iPSCs). (C) Development of therapies using mouse models. (i) The accessibility of the eye and retina allows for delivery of appropriate drugs (e.g. neurotropic factors), gene therapy vectors (viral vectors used for specific gene replacement in recessive disease) and even cells (i′,i″) using intravitreal or subretinal injections/ transplantation in mice as well as in larger animal models. Surgical manipulation of the mouse retina is complicated by the small size of the eye and large lens. (i′) *In vivo* images of cell transplantation into an albino mouse eye. Human embryonic-stem-cell-derived retinal pigment epithelium (hESC-RPE) cells can be transplanted into the mouse eye via transvitreal (left panel; green arrow) or subretinal (right panel; green arrow indicates the location of cell grafts) transplantation. (i″) Histological (left panel) and immunofluorescence (right panel) images of an albino mouse eye after cell transplantation, demonstrating survival and integration of the hESC-RPE cells in the mouse retina. (Left panel) Transplanted pigmented hESC-RPE cells integrate as a monolayer (black staining) in the mouse retina. Inset and arrow indicate transition from host albino cells to grafted cells in the subretinal space. (Right panel) hESC-RPE cells are positive for both the human nuclear antigen (HuNu; green) and premelanosome 17 (PMEL17; red; a typical marker for RPE). (ii) Delivery of gene therapy vectors into photoreceptors or RPE cells will lead to the expression of the appropriate protein and facilitates rescue of function and phenotype.

The eye and especially the retina have become the ‘testing ground’ for novel therapeutic modalities for neurodegenerative diseases by virtue of their accessibility, small size, and the ability to apply multiple techniques to assess structural and functional integrity. After huge success in gene discovery and in the generation of excellent model systems, first-of-their-kind gene- and cell-based therapies are often launched in the eye, targeting retinal disease (Fig. 5). Currently, application of such novel therapies in humans necessitates preclinical testing in animal models in order to prove safety and efficacy, and the most widely used are mouse models of disease.

## Gene therapy

Gene replacement/augmentation therapy relies on the delivery of a normal copy of the defective gene to restore function. Currently, in clinical application, viral vectors are used to transduce the target cells. Pioneering gene therapy trials have become possible because of the use of animal models, and AAV vectors have now been successfully used to deliver target gene(s) to the RPE or photoreceptors (Fig. 5C). The first successful clinical example of such gene therapy came from LCA patients with congenital blindness caused by mutations in the *RPE65* gene (Bainbridge et al., 2008; Cideciyan, 2010; Cideciyan et al., 2008; Maguire et al., 2008). The *Rpe65*-KO mouse (Redmond et al., 1998) and a naturally occurring Briard dog model of RPE65 disease were instrumental in developing this treatment, providing the opportunity to examine and prove safety and efficacy of the vectors prior to application in human patients (Acland et al., 2001; Pang et al., 2006).

A better understanding of RPE65 as well as LRAT function in the visual cycle, based on data from mouse models (Van Hooser et al., 2000), has allowed development of a treatment for respective LCA patients based on supplementation of synthetic 9-*cis* retinoid (Koenekoop et al., 2014). Along these lines, a *Mertk*-KO mouse has been used for testing efficacy of gene therapy for another RPE-specific gene that causes severe arRP, and a clinical trial in patients is ongoing (Conlon et al., 2013) (www.clinicaltrials.gov NCT01482195).

The majority of inherited retinal degenerations are caused by mutations in genes that affect photoreceptor function. Indeed, many mouse models have been used to demonstrate efficacy of gene augmentation therapy to correct defects in genes involved in phototransduction (Bennett et al., 1996; Boye et al., 2010; Michalakis et al., 2012; Tan et al., 2009; Wert et al., 2013) or ciliopathy (Chamling et al., 2013; Lopes et al., 2013; Simons et al., 2011). In the *Bbs4*-KO mouse, AAV-mediated *BBS4* delivery was shown to prevent photoreceptor death and maintain nearly normal-appearing ROS by rescuing rhodopsin mislocalization (Simons et al., 2011). Gene therapy can also rescue defects in *Peripherin2*-KO (Schlichtenbrede et al., 2003) and *Aipl1*-KO (Ku et al., 2011; Sun et al., 2010) mice. Treatment for achromatopsia was successful in *CNGA3*-KO and *CNGB3*-KO mice (Carvalho et al., 2011; Pang et al., 2012). The accumulation of lipofuscin pigment A2E in the retina of *Abca4*-KO mice could be corrected by delivering the intact human *ABCA4* gene (Kong et al., 2008). Clinical gene therapy trials have already begun for patients with several photoreceptor diseases, including Stargardt disease and Usher syndrome (www.clinicaltrials.gov NCT01367444 and NCT01505062).

Retinoschisis, which affects retinal architecture, is another disease nearing clinical application, following successful gene therapy in the *Rs1*-KO mouse model (Min et al., 2005; Zeng et al., 2004). Targeted expression of *RS1* in the IS of photoreceptors via AAV vectors was capable of improving structure and function of the retina in this model (Byrne et al., 2014; Park et al., 2009). The potential of gene therapy in the context of hereditary retinal disease is further highlighted by the recent report of treatment in patients with choroideremia, which is characterized by slow degeneration of the photoreceptors, RPE and choroid (see Box 1) (MacLaren et al., 2014). Mutations in *REP1* (Rab escort protein 1) are the cause of choroideremia, and the treatment of patients was made possible after safety and efficacy were shown in a mouse model (Tolmachova et al., 2013).

Studies in mice also suggest that it would be crucial to deliver the normal gene before maturation of the photoreceptors for maximum efficacy of treatment rather than delivering the gene after maturation (Byrne et al., 2014). Early diagnosis and intervention would thus be desirable for the treatment of RDD patients.

Gene replacement therapy in the eye has benefited greatly from the use of AAV-derived vectors that have retinal tropism, allowing significant advances in gene transfer for both preclinical and clinical research (Koerber et al., 2009; Trapani et al., 2014; Vandenberghe et al., 2011). AAV is safe and delivers genes to both photoreceptors and RPE; however, AAV cannot accommodate genes over 5 kb. Therefore, other methods are being explored. For example, a non-viral nanoparticle has been used to deliver RS1 or RPE65 plasmid to the retina (Delgado et al., 2012; Koirala et al., 2013). Lenti- and adenovirus-based vectors are also being investigated for gene delivery to the retina (Yáñez-Muñoz et al., 2006), but these do not seem to be very efficient in transducing photoreceptors (Puppo et al., 2014). The vast majority of these studies have been conducted in mouse models of disease, which have greatly assisted in the development and optimization of treatment strategies.

## Cell-based therapy

Cell-based therapy is being explored in the context of retinal disease, and first-in-human clinical trials have been recently launched by targeting the RPE. These trials were preceded by studies in animal models, particularly in rodents. Human embryonic (Lu et al., 2009), induced pluripotent stem cell (iPSC)-derived (Buchholz et al., 2009), fetal, umbilical-tissue-derived and bone-marrow-derived (Lu et al., 2010) neuronal and retinal progenitors (Tucker et al., 2011) were examined in models of retinal degeneration, delivered in suspension or on a scaffold. The RPE was chosen as the ‘first target’ because cell replacement in this case would not require formation of neuronal connectivity, and because of the involvement of the RPE in AMD and certain retinal diseases (Ramsden et al., 2013) (Fig. 5B,C). The attempts to differentiate cells that display morphological similarities and characteristics of RPE cells have gained momentum (Idelson et al., 2009; Klimanskaya et al., 2004; Vugler et al., 2008), and stem-cell-derived RPE has been transplanted into the subretinal space to slow the degeneration of photoreceptors in rodent models (Lu et al., 2009; Lund et al., 2006). These experiments led the way to the launching of a first-in-human clinical trial in which RPE cells derived from human embryonic stem cells were transplanted into the subretinal space of patients with AMD or Stargardt disease, and initial results appear promising (Schwartz et al., 2012; Schwartz et al., 2014). Alternatively, direct transplantation of stem or progenitor cells has also shown some promise in animal models, presumably through the secretion of trophic factors that rescue dying cells and attenuate degeneration (Otani et al., 2004). The secretory nature of growth factors such as ciliary neurotrophic factor (CNTF) have been exploited for a novel mode of therapy by intraocular implanting of CNTF-releasing encapsulated cells (Sieving et al., 2006).

An ambitious goal for true regenerative cell therapy for RDDs is the transplantation of photoreceptors because the loss of photoreceptors underlies vision loss in RDDs. Once achieved, this would not only attenuate disease progression (as current forms of gene therapy and RPE transplantation allow) but also lead to tissue replacement and/or repair. Photoreceptor replacement would circumvent difficulties associated with gene-based therapy and could potentially be applied to RDDs with genetic causes and even at an advanced stage of disease. Unlike transplantation of RPE or cells that act via trophic effects, photoreceptor replacement would require not only correct localization and integration of the cells but also the formation of functional synaptic connections with the inner retina. This challenge is currently being addressed and tested in mouse models of RDDs. Post-mitotic photoreceptor precursor cells that express GFP driven by the transcription factor NRL and thus are destined to differentiate into rod photoreceptors (Akimoto et al., 2006; Swaroop et al., 2010) have been shown to integrate into the host retina of different RDD rodent models, and, although the efficacy of integration was initially very low, more recent studies were able to improve transplantation efficacy and demonstrate improvement in retinal and/or visual function (MacLaren et al., 2006; Pearson et al., 2012; Yao et al., 2011). Such integration was also achieved when photoreceptor precursors were derived from mouse embryonic stem cells in culture, rather than collected from early postnatal donor mice (Gonzalez-Cordero et al., 2013). In addition, fully mature photoreceptors taken from adult retina could also integrate in wild-type retina, but with limited survival (Gust and Reh, 2011). Human embryonic-stem-cell-derived and patient-specific iPSC-derived photoreceptor precursors have also been transplanted in the mouse retina, although with limited success (Hambright et al., 2012; Tucker et al., 2013). It is important to note that, although transplantation of rod photoreceptors is showing improved efficacy in mouse models, obtaining cone photoreceptor integration and survival remains elusive. It is clear that we still have a long way before photoreceptor transplantation in humans becomes a reality, but mouse models of RDDs will continue to serve as the primary experimental system in which cell therapy of neuronal tissue will be developed.

## Drug discovery

Potential molecular targets for drug therapy are being identified for different retinal disorders (Fig. 5B,C). Vascular endothelial growth factor (VEGF) plays a key role in neovascularization and vascular leakage in diabetic retinopathy (DR) and AMD (Adamis et al., 1994; Kvanta et al., 1996). Anti-VEGF therapy is valuable for the treatment of neovascular AMD and other retinal diseases (Campochiaro et al., 2011; Jo et al., 2014). The Ras GTPase pathway, which functions downstream of VEGF, is active during development of normal or pathological vascular networks. Negative regulation of this pathway by delivery of α-miR-132, a chemically engineered oligonucleotide with a sequence that is complementary to the endogenous microRNA-132, was shown to prevent angiogenic sprouting in the developing mouse eye (Westenskow et al., 2013). Another success for pharmacological intervention has been achieved in the double-KO *Abca4^−/−^; Rdh8^−/−^* mouse, a model for rod and cone degeneration, resembling features of Stargardt disease. Targeted activation or blocking of the G-protein-coupled receptor (GPCR) signaling pathway and direct inhibition of adenylate cyclase by pharmacological compounds seems to improve photoreceptor cell survival, preserve photoreceptor function and attenuate the accumulation of pathological autofluorescent protein deposits produced by degenerating photoreceptors in the retina (Chen et al., 2013).

In summary, the animal models of RDDs have set the initial stage for developing and testing effective treatment paradigms such as gene therapy, and cell-, drug- and small-molecule-based therapies. Additional animal models of RDDs will expand the scope for developing new treatment for RDDs.

## Conclusions and perspectives

Advances in molecular genetics and particularly next-generation sequencing methods have greatly accelerated the pace of gene discovery for RDDs, and mouse models have been instrumental in deciphering the biology of these debilitating blinding disorders as well as for the development of novel therapeutic modalities. However, although many mouse models have provided novel insights into biochemical and cellular pathways underlying retinal disease, the rodent eye and retina differ from those of the human, and in many instances mouse models do not faithfully replicate the human condition. Basic differences include the fact that rodents are nocturnal and have a rod-dominated retina with only two types of cone photoreceptors (versus three in humans). More importantly, the mouse retina does not have a cone-enriched macula, which is at the center of vision in humans. In addition, dissimilarities in life span and rate of disease progression in mice versus humans can complicate some of the interpretations. Nonetheless, mouse models are currently the leading *in vivo* tool for exploration of disease in general and retinal diseases in particular by virtue of their cost and availability, the ability for genetic manipulation, and the relative ease of their use. An alternative sought-out model for studying RDDs is the zebrafish, because of its phylogenetic proximity to humans. The zebrafish produces large number of embryos, which develop *ex vivo* and are thus amenable to genetic manipulations and experimentation. This makes them an ideal model for high-throughput drug screening. The *ex vivo* development and transparency of zebrafish embryos further enable their use for studying early developmental genes associated with embryonic lethality, which is a bottleneck in mouse models.

With advances in stem-cell and iPSC technology, the first steps to emulate human disease *in vitro* are currently being taken using sophisticated culturing techniques (Eiraku et al., 2011; Nakano et al., 2012). Ultimately, such experimental systems might allow the study of pathogenic mechanisms as well as initial attempts at therapy. However, the need to study interactions within a living mammal will remain, and mouse models of disease will continue to be the mainstay of such efforts. New technologies of genetic manipulation, such as CRISPR-Cas, that allow precision genome editing can be employed to quickly engineer mouse genomes, and it would even be possible to simultaneously alter multiple genes. Such technologies are promising for creating animal models for multigenic complex RDDs and for elucidating pathogenic mechanisms involving gene-gene and gene-environment interactions.

Currently, identification of a gene and mutations associated with RDDs is a relatively easy task because of the availability of tools for genetic analysis. However, to develop treatment for genetic diseases it is necessary to first decode the function of the gene. To increase our knowledge of gene function there is a need to develop better and more efficient tools to target gene manipulation. The subcellular functional analysis of proteins requires more sophisticated technical advancements, such as single-molecule tracking *in vivo* with high-resolution imaging.

The molecular players are relatively better known in rods than in cones. Therefore, future research should focus more in this direction given the crucial role of cones in human vision. The photoreceptors heavily rely upon trafficking modules, but their identity and function is not well understood. Further research in this area can lead to new modes of drug delivery for RDDs. The photoreceptor transplantation treatment for RDDs is limited by the inability of photoreceptors to properly form synaptic connections, because the development, maintenance and function of photoreceptor synapses are poorly understood. Additional investigations are also required to elucidate complex interactions among retinal neurons and supportive retinal Müller glia. We are confident that model organisms, especially mice, will continue to provide original and valuable insights into the biology, disease and therapy of the retina.

## Supplementary Material

Supplementary Material
