# Supplementary material for: Biology and therapy of inherited retinal degenerative disease: insights from mouse models
Source: Dis Model Mech. 2015 Feb;8(2):109–29. doi: 10.1242/dmm.017913 (PMC4314777; doi:10.1242/dmm.017913)
Supplement: Supplementary Material [file supp_8_2_109__index.html]

Biology and therapy of inherited retinal degenerative disease: insights from mouse models — Supplementary Material 

# Biology and therapy of inherited retinal degenerative disease: insights from mouse models

## DMM017913 Supplementary Material

**Files in this Data Supplement:**

- **Supplementary Material**
