# Supplementary material for: Biology and therapy of inherited retinal degenerative disease: insights from mouse models
Source: Dis Model Mech. 2015 Feb;8(2):109–29. doi: 10.1242/dmm.017913 (PMC4314777; doi:10.1242/dmm.017913)
Supplement: Supplementary Material [file supp_8.2.109_DMM017913.pdf]

Supplementary Table S1. Mouse mutants with retinal phenotype maintained at The Jackson Laboratory (http://jaxmice.jax.org/index.html).

Model	Mode of inheritance	Gene	Chr.	Phenotype	Strain/Stock
**Spontaneous mouse mutants**					
rd1	AR	*Pde6b*	5	early onset, severe retinal degeneration	B6.C3-*Pde6b*^{rd1} *Hps4*^{le}/J
pcd	AR	*Agtpbp1*	13	slower retinal degeneration associated with Purkinje cell degeneration (*pcd*)	B6.BR-*Agtpbp1*^{pcd}/J
nr	AR	UN	8	progressive retinal degeneration with hyperactive ataxic behavior (nervous-nr)	BALB/cByJ-*nr*/J
Rd2	AD	*Prph2*	17	slow progressive retinal degeneration (rds)	C3A.Cg-*Pde6b*$^{+}$ *Prph2*^{Rd2}/J
rd3	AR	*Rd3*	1	retinal degeneration, beginning at 3 weeks of age	B6.Cg-*Rd3*^{rd3}/Boc
Rd4	AR	*Gnb1*	4	autosomal dominant retinal degeneration	STOCK In(4)56Rk *Rd4*/J
rd5	AR	*Tub*	7	retinal degeneration, hearing loss and late-developing obesity	B6(Cg)-*Tub*^{tub}/J
mnd	AR	*Cln8*	8	early onset retinal degeneration with a late-onset progressive motor neuron degeneration (mnd)	B6.KB2-*Cln8*^{mnd}/MsrJ
rd6	AR	*Mfrp*	9	small, white retinal spots and	B6.C3Ga-*Mfrp*^{rd6}/J

				progressive photoreceptor degeneration	
rd7	AR	*Nr2e3*	9	retinal spots and progressive photoreceptor degeneration	B6.Cg-*Nr2e3*^{rd7}/J
rd8	AR	*Crb1*	1	focal photoreceptor degeneration	STOCK *Crb1*^{rd8}/J
rd9	XR	*Rpgr*	X	progressive retinal white spotting and degeneration	C57BL/6J-*Rd9*/*Boc*
rd10	AR	*Pde6b*	5	early onset, mild retinal degeneration	B6.CXB1-*Pde6b*^{rd10}/J
rd11	AR	*Lpcat1*	13	retinal degeneration with white retinal vessels at 4 weeks of age	B6.Cg-*Lpcat1*^{rd11}/Boc, B6.Cg-*Lpcat1*$^{rd11\text{-}2J}$/Boc
rd12	AR	*Rpe65*	3	poor ERG response and late onset retinal degeneration[13]	B6(A)-*Rpe65*^{rd12}/J
rd14	AR	*Dcc*	18	slow retinal degeneration with white retinal spots	B6.129S7 Il1r1<tm1Imx>/J-*rd14*
rd15	AR	UN	7	retinal degeneration with retinal outer plexiform dystrophy	B6.Cg-*rd15*
rd16	AR	*Cep290*	10	early onset retinal degeneration	B6.Cg-*Cep290*^{rd16}/Boc
rd17	AR	*Gnat1*	9	poor rod ERG response and slow retinal degeneration	B6.Cg-*Gnat1*^{rd17}
cpfl1	AR	*Pde6c*	19	cone photoreceptor function loss-1	B6.CXB1-*Pde6ccpfl1*/J
Cpfl2	AD	UN	3	cone photoreceptor function loss-2 with white retinal spots	B6CBA-Tg(HDexon1)61Gpb/1J

cpfl3	AR	*Gnat2*	3	cone photoreceptor function loss-3	B6.Cg-*Gnat2cpfl3*/Boc
Cpfl4	AD	UN	17	cone photoreceptor function loss-4	B6CBA-Tg(HDexon1)62Gpb/2J
cpfl5	AR	*Cnga3*	1	cone photoreceptor function loss-5	B6.Cg-*Cnga3*^{cpfl5}
cpfl6	AR	*Hcn1*	13	cone photoreceptor function loss-6	B6;129-*Hcn1*^{tm2Kndl}/J, B6.129S-*Hcn1*^{tm2Kndl}/J
cpfl7	AR	UN	19	cone photoreceptor function loss-7	B6-*cpfl7* (*nm3479)*
nob2	XR	*Cacna1f*	X	anatomical and functional abnormalities (no b-wave-2) in the outer retina	AXB6/PgnJ
nob3	AR	*Grm6*	11	retinal functional abnormalities (no b-wave-3)	B6.B10(D2)-*Grm6*^{nob3}/Boc
arrd2	AR	*Mdm1*	10	age-related retinal degeneration-2	B6.Cg-*Mdm1*^{arrd2}
Krd	AD	*Pax2*	19	kidney and retinal defects	C3.BLiA *Pde6b*$^{+}$-*Krd*/J
Bst	AD	*Rpl24*	16	optic atrophy 1	C57BLKS-*Rpl24*^{Bst}/J
rd18	AR	*Tulp1*	17	retinal degeneration	B6.Cg-*Tulp1*^{rd18}
rd19	AR	*Prom1*	5	retinal degeneraton	B6.Cg-*Prom1*^{rd19}
sedc	AR	*Col2a1*	15	retinoschisis	B6(Cg)-*Col2a1*^{sedc}/GrsrJ
**Chemically induced mouse mutants**					
tvrm64	AR	*Rp1*	1	juvenile onset retinal degeneration	C57BL/6J-*Rp1*^{tvrm64}
nmf12	AR	*Mertk*	2	late onset slow degeneration	C57BL/6J-*Mertk*^{nmf12}

tvrm148	AR	*Rpe65*	3	late onset retinal degeneration	C57BL/6J-*Rpe65*^{tvrm148}
nmf192	AR	*Nphp4*	4	early rapid retinal degeneration	C57BL/6J-*Nphp4*^{nmf192}
Nmf193	AD	*Prph2*	17	retinal degeneration	C57BL/6J-*Prph2*^{Nmf193}/J
nmf247	AR	*Rpgrip1*	14	cone-rod dystrophy	C57BL/6J-Rpgrip1^{nmf247}/J
nmf364	AR	*Pde6b**	5	early rapid retinal degeneration	C57BL/6J-*Pde6b*^{nmf364}
nmf449	AR	*Pde6b**	5	early rapid retinal degeneration	C57BL/6J-*Pde6b*^{nmf449}
Tvrm1	AD	*Rho*	6	light inducible retinal degeneration	C57BL/6J-*Rho*^{Tvrm1}
Tvrm4	AD	*Rho*	6	light inducible retinal degeneration	C57BL/6J-*Rho*^{Tvrm4}
Tvrm144	AD	*Rho*	6	light inducible retinal degeneration	C57BL/6J-*Rho*^{Tvrm144}
tvrm65	AR	*Crx*	7	early rapid retinal degeneration	C57BL/6J-*Crx*^{tvrm65}
tvrm27	AR	*Trpm1*	7	no B-wave	C57BL/6J-*Trpm1*^{tvrm27}
tvrm89	AR	*Myo6*	9	attenuated ERG	C57BL/6J-*Myo6*^{tvrm89}
tvrm84	AR	*Grm1*	10	attenuated ERG	C57BL/6J-*Grm1*^{tvrm84}
nmf246	AR	*Uchl3*	14	juvenile onset retinal degeneration	C57BL/6J-*Uchl3*^{nmf246}
nmf5a	AR	*Pfnd5*	15	early rapid retinal degeneration	C57BL/6J-*Pfnd5*^{nmf5a}
nmf240	AR	*Clcn2*	16	early rapid retinal degeneration	C57BL/6J-*Clcn2*^{nmf240}
nmf223	AR	*Lama1*	17	vitreal fibroplasia, vascular abnormalities	C57BL/6J-*Lama1*^{nmf223}

tvrm124	AR	*Tulp1**	17	early rapid retinal degeneration	C57BL/6J-*Tulp1*^{tvrm124}
nmf282	AR	*Pde6a*	18	early rapid retinal degeneration	C57BL/6J-*Pde6a*^{nmf282}
nmf363	AR	*Pde6a*	18	early rapid retinal degeneration	C57BL/6J-*Pde6a*^{nmf363}
tvrm58	AR	*Pde6a**	18	early rapid retinal degeneration	C57BL/6J-*Pde6a*^{tvrm58}
tvrm32	AR	*Hps1**	18	pigmentation defect	C57BL/6J-*Hps1*^{tvrm32}
**Targeted mouse mutants**					
Abca4^{tm1Ght}	AR	*Abca4*	3	Photoreceptor degeneraton	129S-*Abca4*^{tm1Ght}/J
Abcc6^{tm1Jfk}	AR	*Abcc6*	7	calcified retina	B6.129S1-*bcc6*^{tm1Jfk}/J
Atxn7^{tm1Hzo}	AD	*Sca7*	UN	visual impairment, ataxia	B6.129S7-*txn7*^{tm1Hzo}/J
Bbs1^{tm1Vcs}	AR	*Bbs1*	UN	photoreceptor degeneration	B6.129-*Bbs1*^{tm1Vcs}/J
Bbs2^{tm1Vcs}	AR	*Bbs2*	8	Bardet-Biedl syndrome	B6.129-*Bbs2*^{tm1Vcs}/J
Bbs4^{tm1Vcs}	AR	*Bbs4*	9	Bardet-Biedl syndrome	B6.129-*Bbs4*^{tm1Vcs}/J
Cacna1f^{tm1Sdie}	XR	*Cacna1f*	X	night blindness	B6(Cg)-*Cacna1f*^{tm1Sdie}/J
Cep290^{tm1Jgg}	AR	*Cep290*	10	retinal degeneration	STOCK *Cep290*^{tm1Jgg}/J
Cln3$^{tm1.1Mem}$	AR	*Cln3*	7	retinal degeneration	B6.129(Cg)-*Cln3*$^{tm1.1Mem}$/J
Crx^{tm1Clc}	AD	*Crx*	7	cone-rod dystrophy 2	B6.129-*Crx*^{tm1Clc}/J
Fzd4$^{tm2.1Nat}$	AR	*Fzd4*	7	Vitreoretinopathy	B6;129-*Fzd4*$^{tm2.1Nat}$/J
Lrp5^{tm1Dgen}	AR	*Lrp5*	19	Vitreoretinopathy	B6.129P2-*Lrp5*^{tm1Dgen}/J

Mkks^{tm1Vcs}	AR	*Mkks*	2	retinal degeneration	B6.129-*Mkks*^{tm1Vcs}/J
Ndp^{tm2Nat}	XR	*Ndp*	X	Norrie disease	B6;129-*Ndp*^{tm2Nat}/J
Nrl^{tm1Asw}	AR	*Nrl*	14	retinal degeneration	B6.129-*Nrl*^{tm1Asw}/J
Opn1mw$^{tm1(OPN1LW)Nat}$	XR	*Opn1mw*	X	abnormal vision	B6.129-*Opn1mw*$^{tm1(OPN1LW)Nat}$/J
Prom1$^{tm1(cre/ERT2)Gilb}$	AR	*Prom1*	5	retinal degeneration	B6N;129S-*Prom1*$^{tm1(cre/ERT2)Gilb}$/J
Rho$^{tm1.1Kpal}$	AD	*Rho*	UN	retinal degeneration	B6.129S6(Cg)-*Rho*$^{tm1.1Kpal}$/J
Rom1^{tm1Mci}	AR	*Rom1*	19	retinal degeneration	STOCK *Rom1*^{tm1Mci}/J
Sag^{tm1Jnc}	AR	*Sag*	1	retinal degeneration	STOCK *Sag*^{tm1Jnc}/J
Timp3$^{tm1.1(KOMP)Vlcg}$	UN	*Timp3*	10	fundus dystrophy	B6N(Cg)-*Timp3*$^{tm1.1(KOMP)Vlcg}$/J
Ttpa^{tm1Far}	UN	*Ttpa*	4	retinal degeneration	B6.129S4-*Ttpa*^{tm1Far}/J
Tulp1^{tm1Pjn}	AR	*Tulp1*	17	retinal degeneration	B6.129X1-*Tulp1*^{tm1Pjn}/Pjn
Ush1c^{tm1Xzl}	AR	*Ush1c*	7	Usher Syndrome, Type Ic	B6.129-*Ush1c*^{tm1Xzl}/Kjn

Chr., chromosome; AD, autosomal dominant; AR, autosomal recessive; UN, unknown; XD, X-linked dominant; XR, X-linked recessive. * Established by complementation testing.
